# Supplementary material for: Genetic Analysis of an F2 Population Derived from the Cotton Landrace Hopi Identified Novel Loci for Boll Glanding
Source: Int J Mol Sci. 2024 Jun 27;25(13):7080. doi: 10.3390/ijms25137080 (PMC11241279; doi:10.3390/ijms25137080)
Supplement: Supplementary file 1 [file ijms-25-07080-s001.zip › ijms-3047578-supplementary/Supplementary files/S1 Figure.pptx]

## Slide 1
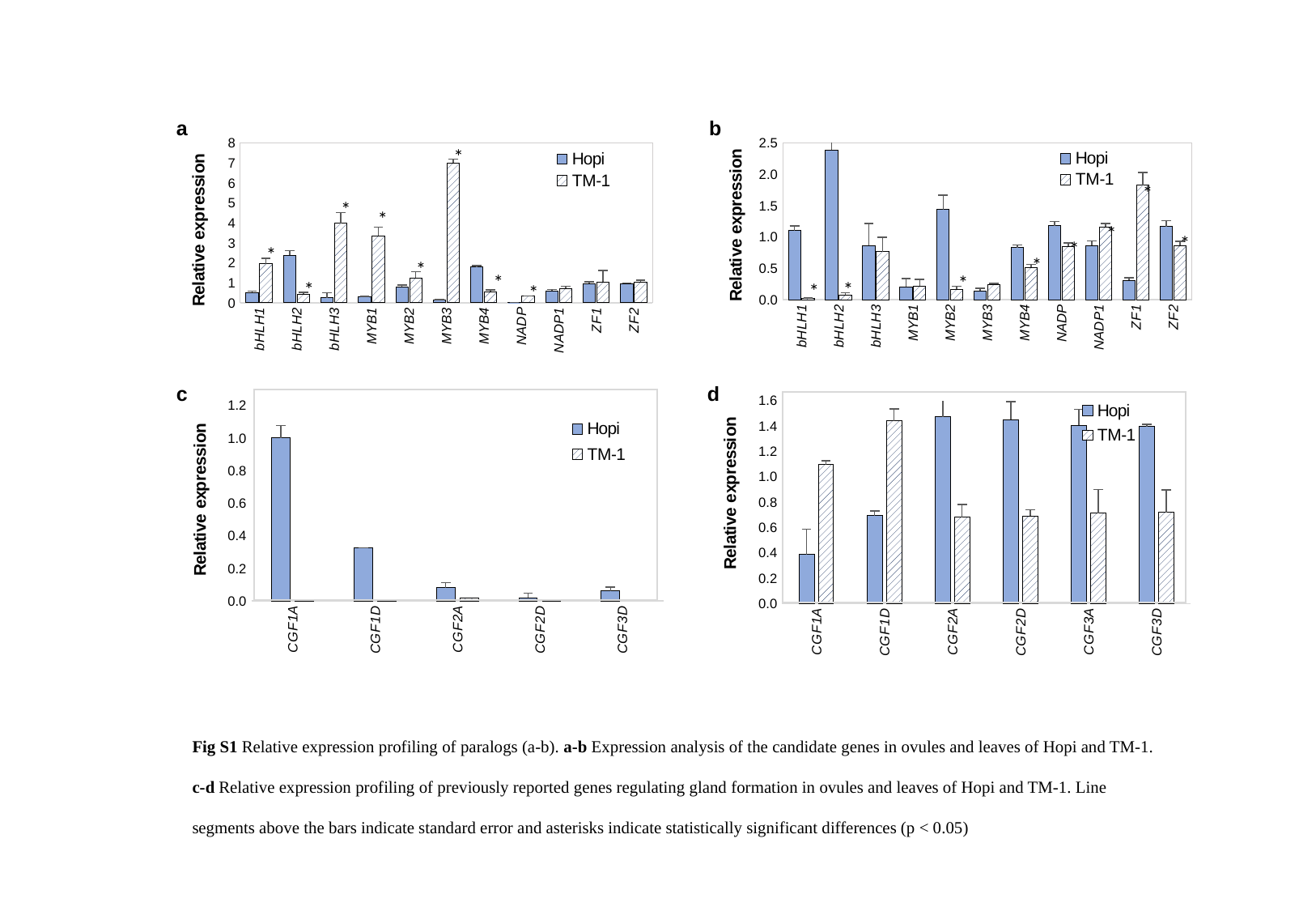

a
b
### Chart
| Category | Hopi | TM-1 |
|---|---|---|
| bHLH1 | 0.51545607002351 | 1.94002953530917 |
| bHLH2 | 2.37082178949903 | 0.421794672391342 |
| bHLH3 | 0.250924976498215 | 3.98525493139626 |
| MYB1 | 0.299542107446255 | 3.33842880563769 |
| MYB2 | 0.804286055201875 | 1.24333872697694 |
| MYB3 | 0.14295287654997 | 6.99531219052068 |
| MYB4 | 1.7771263724662 | 0.562706184260972 |
| NADP | 0.00820286950626152 | 0.324811167156775 |
| NADP1 | 0.593830281519894 | 0.71900472169364 |
| ZF1 | 0.966793008817778 | 1.0343475706582 |
| ZF2 | 0.951165906544846 | 1.03706885944696 |
### Chart
| Category | Hopi | TM-1 |
|---|---|---|
| bHLH1 | 1.09904241670244 | 0.0248127825853613 |
| bHLH2 | 2.37867761115252 | 0.0783317616656956 |
| bHLH3 | 0.861890718201986 | 0.764804363674603 |
| MYB1 | 0.203448054720842 | 0.216597485004029 |
| MYB2 | 1.43290683808512 | 0.167528727906315 |
| MYB3 | 0.141895251012617 | 0.243648680771403 |
| MYB4 | 0.828829903681668 | 0.512402998580106 |
| NADP | 1.17670686671691 | 0.849829323075227 |
| NADP1 | 0.8638627968534 | 1.15759123282363 |
| ZF1 | 0.310108156881458 | 1.82056321553805 |
| ZF2 | 1.16480413071187 | 0.858513438983817 |c
d
### Chart
| Category | Hopi | TM-1 |
|---|---|---|
| CGF1A | 1.0 | 0.0 |
| CGF1D | 0.32522 | 0.0 |
| CGF2A | 0.08064 | 0.02034 |
| CGF2D | 0.0186 | 0.0105 |
| CGF3D | 0.061 | None |
### Chart
| Category | Hopi | TM-1 |
|---|---|---|
| CGF1A | 0.38768 | 1.09418 |
| CGF1D | 0.69407 | 1.44078 |
| CGF2A | 1.46998 | 0.68028 |
| CGF2D | 1.44799 | 0.69061 |
| CGF3A | 1.40568 | 0.7114 |
| CGF3D | 1.3936 | 0.71757 |
*
*
*
*
*
*
*
*
*
*
*
*
*
*
*
*
Fig S1 Relative expression profiling of paralogs (a-b). a-b Expression analysis of the candidate genes in ovules and leaves of Hopi and TM-1.
c-d Relative expression profiling of previously reported genes regulating gland formation in ovules and leaves of Hopi and TM-1. Line segments above the bars indicate standard error and asterisks indicate statistically significant differences (p < 0.05)
